# What are Altmetrics and why would anyone be interested?

**DOI:** 10.1002/nop2.55

**Published:** 2016-05-04

**Authors:** Roger Watson

Go to the landing page of *Nursing Open* and click on an issue and then on an article and above the tabs that take you to the content of the article you will see a coloured indicator labelled ‘AM score’ and, in some cases, a number next to it. That number is the Altmetric^®^ score; Altmetrics being a contraction of ‘alternative metrics’. This score indicates the extent to which the article has received attention on social media sites, such as Twitter^®^ and on blogs. If you hover over the ‘AM score’, you will get information such as the number of times the link to the article has been mentioned on, for example, Twitter^®^ or Facebook^®^. If you click on the indicator, then you will open up a more detailed webpage showing a map of the World and the countries where the mentions came from filled in and also the demographic breakdown of those mentioning the article. You may be puzzled about why, sometimes, the number of mentions does not equal the score which may be lower or higher than the number of mentions. This is because Altmetric^®^ scores are weighted. For example, a mention on a blog is worth five points compared with a mention on Twitter^®^, which is worth one point. Some mentions are worth less than 1, for example, YouTube^®^ and LinkedIn^®^.

Above is an explanation of what the Altmetric^®^ score is, but what should we be interested? As an editor, I am interested to see how much impact what we publish has; likewise, the publishers wish to know. Traditional metrics, such as total citations and impact factors remain important measures of impact, or influence, on the scientific community but they are flawed. Altmetrics are not perfect either but they add to the information we have about how much attention as article is receiving and their use acknowledges the power of social media. The aim of most authors was to have their work read and cited in other works. This, at least, indicates that their work has been found useful – if not necessarily agreed with – and has helped someone else to frame their work or their arguments. Towards that end, mentions on social media are known to predict and possibly increase citations to articles (Eysenbach 2011, Knight 2014), therefore, monitoring Altmetrics – which are almost instant – can be useful in indicating the extent to which an article is likely to be cited. It is also an explanation of why we have a blog and a Twitter^®^ site at *Nursing Open* and why more authors are turning to social media to promote their work.
